# Community health workers to improve adherence to anti‐seizure medication in rural South Africa: Is it cost‐effective?

**DOI:** 10.1111/epi.16756

**Published:** 2020-11-25

**Authors:** Ryan G. Wagner, Fredrik Norström, Melanie Y. Bertram, Stephen Tollman, Lars Forsgren, Charles R. Newton, Lars Lindholm

**Affiliations:** ^1^ Studies of Epidemiology of Epilepsy in Demographic Surveillance Systems (SEEDS) – INDEPTH Network Accra Ghana; ^2^ MRC/Wits Rural Public Health & Health Transitions Research Unit (Agincourt) School of Public Health Faculty of Health Sciences University of the Witwatersrand Johannesburg South Africa; ^3^ Department of Epidemiology and Global Health Umeå University Umeå Sweden; ^4^ Department of Clinical Sciences, Neurosciences Umeå University Umeå Sweden; ^5^ World Health Organization Geneva Switzerland; ^6^ KEMRI/Wellcome Trust Research Programme Centre for Geographic Medicine Research – Coast Kilifi Kenya; ^7^ Department of Psychiatry University of Oxford Oxford UK

**Keywords:** cost‐effectiveness analysis, economic, epilepsy, model, treatment

## Abstract

**Objective:**

Epilepsy is a common, chronic neurological disorder that disproportionately affects individuals living in low‐ and middle‐income countries (LMICs), where the treatment gap remains high and adherence to medication remains low. Community health workers (CHWs) have been shown to be effective at improving adherence to chronic medications, yet no study assessing the costs of CHWs in epilepsy management has been reported.

**Methods:**

Using a Markov model with age‐ and sex‐varying transition probabilities, we determined whether deploying CHWs to improve epilepsy treatment adherence in rural South Africa would be cost‐effective. Data were derived using published studies from rural South Africa. Official statistics and international disability weights provided cost and health state values, respectively, and health gains were measured using quality adjusted life years (QALYs).

**Results:**

The intervention was estimated at International Dollars ($) 123 250 per annum per sub‐district community and cost $1494 and $1857 per QALY gained for males and females, respectively. Assuming a costlier intervention and lower effectiveness, cost per QALY was still less than South Africa's Gross Domestic Product per capita of $13 215, the cost‐effectiveness threshold applied.

**Significance:**

CHWs would be cost‐effective and the intervention dominated even when costs and effects of the intervention were unfavorably varied. Health system re‐engineering currently underway in South Africa identifies CHWs as vital links in primary health care, thereby ensuring sustainability of the intervention. Further research on understanding local health state utility values and cost‐effectiveness thresholds could further inform the current model, and undertaking the proposed intervention would provide better estimates of its efficacy on reducing the epilepsy treatment gap in rural South Africa.

**Key Points**

- Epilepsy disproportionately affects low‐ and middle‐income countries (LMICs) where shortages of trained health professionals and high epilepsy treatment gaps concurrently exist
- Task shifting care through the employ of community health workers (CHWs) has been shown to improve outcomes for a number of chronic conditions in LMIC settings
- No study has determined the cost‐effectiveness of task‐shifting the provision of follow‐up epilepsy care to CHWs
- Undertaking a health economic evaluation, we found that employing CHWs in rural South Africa is a cost‐effective intervention to reduce the epilepsy treatment gap

## 1 INTRODUCTION

Epilepsy is one of the most severe, chronic neurological conditions globally and disproportionately affects people living in low‐ and middle‐ income countries (LMICs), where up to 80% of the roughly 50 million people with epilepsy reside.^1^, ^2^ A study from rural South Africa found the adjusted prevalence of active convulsive epilepsy to be 7.0 per 1000 individuals, which represents only a proportion of all epilepsies.^3^ The epilepsy treatment gap, defined as the number of people with epilepsy either not on treatment or on inadequate treatment is high; with a meta‐analysis finding the epilepsy treatment gap to be at least 50% in LMICs and a higher treatment gap in rural areas when compared to urban areas.^4^ A number of factors have been suggested for the observed treatment gap including traditional beliefs, lack of access to anti‐seizure medications (ASMs), inadequate medical care facilities and providers, lack of training, and cost of treatment.^4^, ^5^

Non‐adherence to ASMs has been linked to increased seizure frequency, higher healthcare costs, and greater mortality.^6^, ^7^ This is in addition to poorer educational outcomes, greater risk of physical injuries, depression and anxiety, and higher levels of stigma experienced by people with epilepsy.^8^, ^9^ Improving adherence to ASMs, thereby reducing seizure frequency, will likely reduce the mortality and improve the quality of life in people with epilepsy.

LMICs, including those in Africa, are faced with a shortage of trained medical professionals. Within these countries, rural areas have fewer medical personnel to cater for a population that often carries a higher burden of disease. Community health workers (CHWs), individuals who lack formal professional tertiary education though are provided with job‐related training, have been introduced at the community and primary health level in an attempt to address this void. A Cochrane review found that CHWs increased the uptake of immunizations and breast feeding, improved tuberculosis treatment outcomes, and reduced child morbidity and mortality when compared to general care.^10^ Studies have also shown CHWs to be cost‐effective for the treatment and follow‐up of other chronic conditions, including human immunodeficiency virus (HIV).^11^

Task shifting in the provision of epilepsy care has also been proposed and undertaken, with earlier studies from Africa focused on training primary healthcare (PHC) nurses to provide care. A study from Zimbabwe found that training nurses at primary health facilities in epilepsy care and referral resulted in a 74% increase in patient recruitment and a 17% increase in ASM adherence, although it did not find a reduction in seizure frequency.^12^ However, a separate, large retrospective study using Medicaid claims data from the United States did find seizure frequency to be associated with adherence.^7^ Nurse‐run PHC epilepsy clinics in rural Ethiopia that were integrated into the healthcare system were found to result in a reduction of seizure frequency in >90% of patients and, after 2 years, 73% of people with epilepsy were still under follow‐up.^13^

More recently, efforts have shifted to employing CHWs to provide epilepsy care, with the view that CHWs tasked with following people with epilepsy are likely to have a positive effect on treatment adherence and seizure reduction.^5^ A study from rural Guinea‐Bissau found that employing a community‐based rehabilitation program, largely aligned with the World Health Organization's mental health Gap Action Programme (mhGAP), decreased seizure frequency by 88.8% after 15 months of the program. The authors further suggest that the intervention is cost‐effective; however, they do not present a formal cost‐effectiveness analysis.^14^ A clinical trial currently underway in northern Nigeria is examining the efficacy of using community health extension workers to reduce the epilepsy treatment gap in children.^15^

Although CHWs have been shown to be a cost‐effective intervention for delivering a number of important health services,^11^ no study has formally analyzed the cost‐effectiveness of a CHW intervention aimed at reducing the epilepsy treatment gap. In this study, we modeled the cost‐effectiveness of training CHWs in rural South Africa to educate community members and traditional healers about epilepsy and epilepsy treatment options and regularly visit people with epilepsy to improve adherence and initiate referral when needed, in line with the South African government's envisaged role of CHWs.^16^ We compare this intervention against current practice.

## 2 METHODS

A health economic evaluation was performed using a four‐state Markov model with age‐ and sex‐varying disease transition probabilities to estimate the effects of introducing a CHW‐led intervention aimed at educating local community leaders and patients about epilepsy and improving ASM adherence and referral pathways. Recently it was found that 68% of people with active convulsive epilepsy reported taking treatment and 71% had any detectable level of ASMs in their blood, with an epilepsy treatment gap of 63%.^17^ Even with these comparably high levels of adherence, 57% still experienced seizures at least once per month.

### 2.1 Target population and characteristics

Data were derived from the Agincourt sub‐district of the Bushbuckridge Health District, located 500 km northeast of Johannesburg, South Africa. The Agincourt sub‐district, fully covered by the Agincourt Health and Socio‐demographic Surveillance System (HDSS) (http://www.agincourt.co.za) comprises 31 contiguous villages located on 450 km^2^ of semi‐arid scrubland. In 2016, the sub‐district comprised nearly 115 000 individuals living in roughly 22 000 households. A former homeland during *Apartheid*, the Agincourt population experiences high levels of unemployment, resulting in high levels of labor migration. HIV and tuberculosis (TB) substantially contribute to the disease burden both in Agincourt and nationally, yet noncommunicable diseases, including cardiometabolic disease are becoming more prominent as the population ages.

The healthcare needs of the population are served by seven primary PHC clinics staffed by government nurses, which offer free basic outpatient health services, including ASMs (phenytoin, carbamazepine, sodium valproate, phenobarbitone); two larger government PHC health centers offering 24‐hour care; and three district hospitals.

Efforts are currently underway by the South African Department of Health in the Agincourt sub‐district to establish ward‐based outreach teams (WBOTs). Comprising a professional nurse, an environmental health officer, health promoters, and 6 to 10 CHWs, these teams seek to improve access and health outcomes by taking health services to the community. These health services include basic health screening, attending minor ailments, controlling chronic disease, undertaking family planning and mother and child care and referring to clinics.^18^ The WBOT intervention will be evaluated using routine health indicators including antenatal care coverage and attendance, PHC utilization rates, and immunization coverage.^19^

### 2.2 Model description

**Figure 1:**
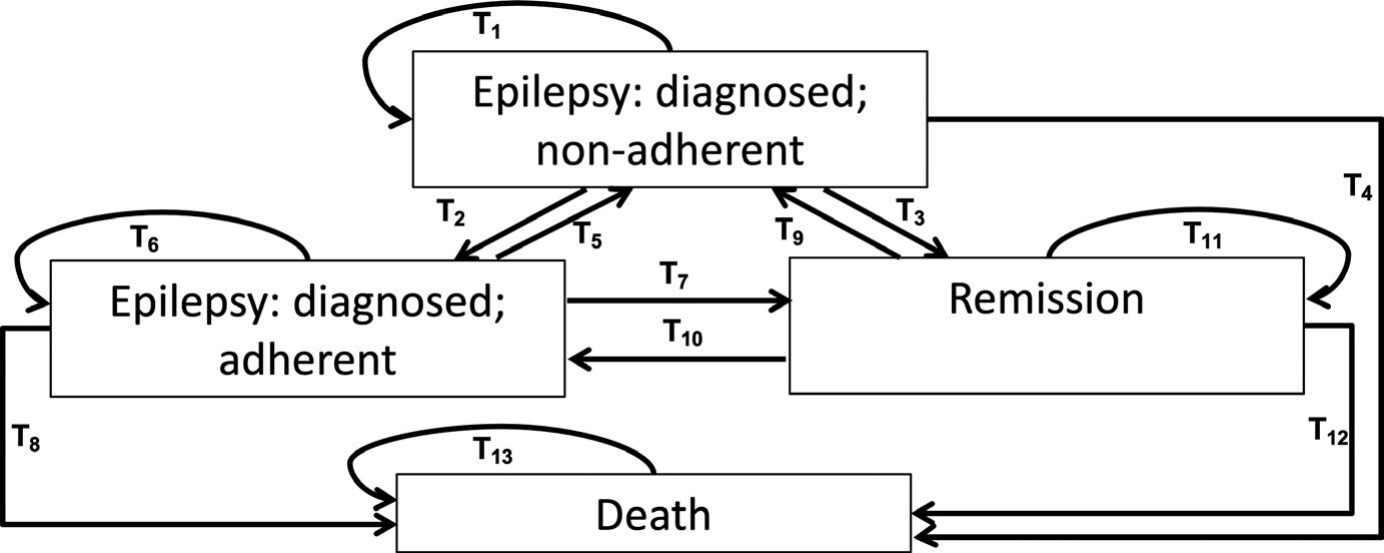
Four‐state Markov model used in this study to estimate the cost‐effectiveness of community health workers (CHWs) on improving the adherence to anti‐epileptic drugs (AEDs)

A Markov model with four health states, representing the chronicity and potential transitions of a person with epilepsy over time, was developed. This type of modeling was chosen given the chronic nature of epilepsy and the possibility of “returning” to a previous state (moving from adherent to nonadherent back to adherent over a period of time).^20^ The model developed can be found in Figure 1, with four health states represented in the rectangular boxes. The health states include: (A) being diagnosed with epilepsy and nonadherent to treatment; (B) being diagnosed with epilepsy and adherent to treatment; (C) remission; and (D) death (Figure 1). The model assumes that the person with epilepsy has already been diagnosed, hence the individual “enters” the model in either the “epilepsy: diagnosed; adherent” or the “epilepsy: diagnosed; nonadherent” state. The model was run for 100 cycles, with the cycle time representing 1 year, taking a life‐perspective approach.

### 2.3 Transition probabilities

The lines between the states represent the transition probabilities, or the rates at which individuals from one health state are likely to “transition” to another health state. The transition probability refers to the conditional probability of the individual transferring to the same or another state during the cycle.^21^ For example, the transition from epilepsy: diagnosed; adherent to remission (T_7_ in Figure 1) represents the rate of remission. Published remission and mortality figures from the Agincourt sub‐district were used to derive the transition probabilities within the model, with remission defined as not being on ASM and being seizure free for 1 year.^22^The model adjusted mortality and remission rates by age.^22^ Remission rates from adherent and nonadherent states to remission were modeled as the same rate, varying by age and sex (Table 1). It was estimated that the mortality rates for people who were nonadherent were 2.5 times greater than mortality rates recently published from Agincourt, whereas for those who were adherent mortality rates were 50% lower than those reported (Table 1). This equates to a mortality 5 times greater for those that are nonadherent vs those who are adherent, a figure comparable to unadjusted figures published from the United States.^6^ Age‐ and sex‐varying transition probabilities from remission to death were estimated as the background mortality rate in the Agincourt sub‐district (Table 2). Age‐varying relapse figures were derived from an earlier article by Annegers and colleagues^23^ (Table 2), with the current model assuming that 50% of relapsing individuals would transition from remission to adherent and 50% to nonadherent.

**TABLE 1:** Mortality rates in adherent and nonadherent individuals with epilepsy by age and remission rates by age and sex

Age band	Mortality in nonadherent (T_4_)	Mortality in adherent (T_8_)	Remission (T_3_ & T_7_)	
			Male	Female
0‐5	0.030	0.006	0.160	0.305
6‐12	0.054	0.011	0.033	0.066
13‐18	0.019	0.004	0.045	0.004
19‐28	0.043	0.009	0.093	0.013
29‐49	0.060	0.012	0.000	0.031
50+	0.074	0.015	0.020	0.065

**TABLE 2:** Background mortality rates by age and sex and relapse rates by age

Age band	Overall mortality (T_12_)		Rate of relapse (T_9_ & T_10_)
	Male	Female	
0	0.050	0.040	0.000
1‐4	0.006	0.005	0.000
5‐9	0.001	0.001	0.000
10‐14	0.001	0.001	0.002
15‐19	0.001	0.002	0.002
20‐24	0.004	0.006	0.004
25‐29	0.010	0.012	0.004
30‐34	0.017	0.016	0.005
35‐39	0.021	0.016	0.005
40‐44	0.028	0.016	0.007
45‐49	0.025	0.016	0.007
50‐54	0.024	0.020	0.009
55‐59	0.029	0.016	0.009
60‐64	0.052	0.019	0.010
65‐69	0.056	0.025	0.010
70‐74	0.066	0.024	0.012
75‐79	0.071	0.036	0.012
80‐84	0.099	0.067	0.012
85‐89	0.118	0.107	0.012
90‐94	0.190	0.091	0.012
95+	0.190	0.170	0.012

### 2.4 Health state valuations

Health state valuations (1 = no impairment; 0 = dead) were determined by subtracting from one the disability weight published in the 2010 Global Burden of Disease (2010 GBD) study,^24^ with modifications taking into account the seizure frequency in those who were adherent (Table 3). The 2010 GBD reported differing disability weights (DWs) related to whether an individual was on treatment or not as well as seizure frequency, which were derived from a survey of more than 40 000 respondents from a range of settings and countries.^24^

**TABLE 3:** Health state utility values and corresponding derivations from the 2010 Global Burden of Disease study

Health state utility values	QALY	Derivation
State A: Diagnosed; nonadherent	0.58	1−0.42 (untreated epilepsy)
State B: Diagnosed; adherent	0.8292	1−(60%*0.072 (treated seizure‐free) + 40%*0.319 (treated with seizures)
State C: Remission	0.928	1−0.072 (treated seizure‐free)
State D: Death	0	0 (no utility)

### 2.5 Intervention description

In line with South African Department of Health guidelines, individuals with secondary school qualifications (grade 12 level) would be eligible to apply to become a CHW.^25^ CHWs will undergo an intensive and rigorous training led by provincial neurologists and district clinicians and supported by professional nurses. Given the paucity of neurologists in this rural context and to ensure adequate capacity for support by local healthcare professionals, it is envisaged that a “training of trainers” cascade model would be employed where neurologists would train local clinicians and professional nurses who would then train the CHWs, as has been done recently in neighboring Mozambique.^26^ Groups such as Epilepsy South Africa (www.epilepsy.org.za) will also be invited to provide training on the social aspects of epilepsy. The training material would be largely derived from the World Health Organization's mental health care GAP (mhGAP) and the South African Primary Health Care guidelines, and as done in a similar ongoing intervention in northern Nigeria, supplemented with modified epilepsy nurse training material from the United States.^15^

Monitoring and evaluation of the performance of the intervention is central to ensuring its success of the intervention. Similar to the structure employed by the WBOT intervention, key indicators will be recorded by both the CHWs and the PHC facilities and regularly checked by the Program Coordinator. These indicators will include number of visits to people with epilepsy made and experienced number of seizures, number of referrals to PHC facilities, and number of community meetings held.

Besides annual community meetings held to inform community members and traditional healers on epilepsy, CHWs will visit people with epilepsy once every 3 months to assess adherence. During these visits, the CHW will review patient‐held seizure diaries, inquire about seizure frequency, provide psychological support, and refer the patient to the primary healthcare facilities if seizures are uncontrolled or comorbidities are present. Furthermore, CHWs will regularly meet with community leaders, including school principals, to provide education on epilepsy. It is estimated that four CHWs will be able to make eight visits per day and be able to visit all people with epilepsy within the sub‐district in a 3‐month period, assuming a 1.5% prevalence of active epilepsy, a reasonable estimate of the prevalence of active epilepsy.^3^ Annual costs for the intervention are found in Table 4.

**TABLE 4:** Cost associated with intervention of CHWs for the improvement of ASM adherence

Intervention Costs	in ZAR (per annum)
*Salaries*	
4 Community Health Workers	240 000
1 Program Coordinator	400 000
*Training*	
Trainer salary	5000
Room & equipment rental	500
*Consumables*	
Cell phone & airtime	5000
Stationary	3000
Pamphlets	2500
Local transport	8320
Total	664 320

### 2.6 Effectiveness data

It is anticipated that the intervention will result in 90% adherence levels within 2 years of implementation, which will lead to seizure freedom in 60% and a reduction in seizures in an additional 40% of people with epilepsy. These assumptions are derived from an understanding that 70% of individuals with epilepsy can be adequately controlled with pharmacotherapy^27^ and from previous studies from Zimbabwe and Ethiopia, which employed PHC nurses to lead epilepsy care clinics and a more recent study from rural Guinea Bissau that found a reduction of seizures in 88.8% of patients after 15 months of a community‐based rehabilitation program.^12^, ^13^, ^14^ Interventions using CHWs to improve drug adherence for tuberculosis and HIV have found similar levels of effectiveness as those suggested in this study.^28^, ^29^

### 2.7 Medicine prices

The analysis was performed from a societal perspective and included costs of ASMs, healthcare utilization costs, and cost of lost productivity (Table 5). ASM costs were estimated by calculating the proportion of drugs and drug combinations currently prescribed in the Agincourt cohort of people with epilepsy (unpublished data) multiplied by the cost per unit as reported in the South African Database of Medicine Prices.^30^

**TABLE 5:** Costs associated with various states of Markov model

	in ZAR (per annum)	±15%
*State A: Nonadherence*		
Anti‐epileptic drugs	0	0
Out‐patient clinic visits	650	553‐748
Out‐patient hospital visits	334	284‐384
In‐patient hospital stays (incl. ambulance)	4533	3853‐5213
Lost productivity	10 823	9199‐12 446
Total	16 340	13 889‐18 790
*State B: Adherence*		
Anti‐epileptic drugs	880	748‐1012
Out‐patient clinic visits	1300	1105‐1495
Out‐patient hospital visits	334	284‐384
In‐patient hospital stays (incl. ambulance)	2267	1927‐2606
Lost productivity	3900	3315‐4485
Total	8681	7378‐9983
*State C: Remission*		
No cost	0	
*State D: Death*		
No cost	0	

### 2.8 Healthcare utilization costs and lost productivity

Healthcare utilization costs were derived from the Mpumalanga Department of Health's uniform patient fee schedule.^31^ Cost of lost productivity was estimated using the 2014 South African gross domestic product (GDP) per capita estimate of purchasing power parity international dollar ($) 13 215 and an exchange rate of one USD to 5.39 ZAR,^32^ resulting in a GDP per capita of 71 229 ZAR and a GDP per capita per day of 195 ZAR.

### 2.9 Statistical analysis

An incremental cost‐effectiveness ratio (ICER) was calculated as the difference in costs between the intervention and no intervention scenarios divided by the gain in QALYs due to the intervention. Sensitivity analyses were performed on both the effectiveness of the intervention (−50%) and costs of the intervention (+50%). Due to variability in mortality and remission rates between males and females, the resulting ICER is reported for both males and females.

## 3 RESULTS

A CHW intervention to improve the adherence to ASMs in people with epilepsy was found to cost 664 320 ZAR ($123 250) per annum for a sub‐district, or roughly 443 ZAR ($82) per individual with epilepsy. For males, based on the assumed effect of the intervention and the resulting increased ASM cost due to improved adherence introduced by the CHWs, this intervention will result in 5.90 QALYs gained for an added cost of 47 480 ZAR ($8808), yielding a cost effectiveness ratio of 8053 ZAR ($1494) per QALY gained. For females, the intervention will result in 4.09 QALYs gained at an added cost of 40 969 ZAR ($7601), yielding an ICER of 10 009 ZAR ($1857) per QALY gained. Using the willingness‐to‐pay threshold of $2154 (Afr E region of WHO; www.who.int/choice/costs/CER_thresholds_regions.xls) or roughly 11 610 ZAR, or even the threshold of one times GDP (which was roughly 71 229 ZAR in 2014), this intervention can be considered to be highly cost‐effective using the modeled parameters and costs.

Increasing the cost of the intervention by 50% resulted in a cost per QALY of 11 809 ZAR ($2191) and 15 419 ZAR ($2860) for males and females, respectively. These values fell well below the cost‐effectiveness threshold of one times GDP for South Africa.

The results were sensitive to the effect of the intervention. Reducing the effect of the intervention by 50% resulted in an ICER of 25 437 ZAR ($4719) per QALY gained for males and 35 246 ZAR ($6539) per QALY gained for females; an increase of roughly 3 and 3.5 times cost per QALY, respectively. These ICER values are still below the cost‐effectiveness threshold of one times GDP for South Africa.

## 4 DISCUSSION

A number of cost‐effective analyses have previously been undertaken exploring interventions for the treatment of epilepsy, including pharmacological interventions,^33^, ^34^ surgical and diagnostic interventions,^35^, ^36^ and a dietary intervention,^37^ with a 2017 systematic review summarizing these analyses.^38^ Yet to our knowledge, no formal cost‐effectiveness analysis has been undertaken to explore the impact of a CHW on increasing adherence to ASMs and thereby reducing the epilepsy treatment gap. In this modeling study, we found the introduction of a CHW to be a cost‐effective intervention in rural South Africa, resulting in a cost per QALY below the conservative threshold of one times GDP per capita.^39^ The cost‐effectiveness threshold value is an arbitrary figure that is thought to represent the cost one is willing to pay to gain one year of healthy life (1 QALY).

Some countries favor the use of graduated cost‐effectiveness threshold, depending on the severity of the condition. Conditions that are considered more disabling have a higher threshold value than conditions that are considered less disabling.^40^ Epilepsy can vary in terms of its severity, with severe epilepsy among the most disabling condition globally.^24^ This suggests, using a graduated cost‐effectiveness threshold approach that interventions developed for treating epilepsy can cost more and still maintain their cost‐effectiveness, which would further support our findings.

Our findings that a CHW intervention is cost‐effective are similar to findings from a recent study exploring the use of a CHW to provide education and blood pressure testing in the same context.^41^ Furthermore, the proposed intervention speaks to the need of interventions to be sustainable, embedded in the local context and national strategy.

South Africa is undergoing a PHC re‐engineering and revitalization that speaks to the transitioning disease burden (parallel communicable and noncommunicable disease epidemics) and its ability to address chronic disease treatment and prevention.^42^ CHWs play a key role in the ward‐based outreach team envisaged by the South African Ministry of Health,^43^ filling a void in the understaffed healthcare workforce and contributing to an overburdened PHC system. Although South Africa already has nearly 72 000 trained CHWs nationally, these individuals are primarily focused on infectious diseases (HIV, TB) and child and maternal health care,^43^ with noncommunicable disease management not yet receiving priority.^44^ Serving as a bridge between community members and health facilities, the success of the CHW, and ultimately the modeled intervention, relies on a number of factors including clear supervision and responsibilities,^43^ adequate recognition, support and capacity development, and clear referral pathways.^44^ Discussions with PHC nurses within the Agincourt subdistrict suggest that adherence to ASMs has improved during the 3‐year follow‐up of people with epilepsy as part of the ongoing epilepsy research (N. Machave, personal communication). Introducing the modeled intervention into the Agincourt subdistrict would generate the required data to determine whether the modeled cost‐effective intervention is truly cost‐effective.

### 4.1 Limitations

Models are, inherently, an attempt to simplify complex, real‐world events into simulations that are sufficiently accurate to be useful to describe and evaluate a situation. Models rely on the best available data to populate them and, often, missing parameters are filled in with “expert opinion.” This model is no different: We used the best available, previously published data and expert opinion to model the effects of a CHW intervention on the adherence to ASM treatment for epilepsy. As such, this model is limited by the reliability of the input parameters, including the estimation on the effectiveness of the intervention. It is possible that certain aspects of the model need to be refined during implementation. The impact that this could have in terms of possibly reduced efficacy or increased cost of the intervention has been, at least, partially addressed by undertaking a sensitivity analysis. The robustness of the cost‐effective findings is supported by the sensitivity analysis. Undertaking an intervention study in rural South Africa, based on the cost‐effective intervention proposed here, and comparing the outcome cost and QALY effects of the real‐world intervention to the findings of this study would be an interesting future direction and could lend credence to the usefulness of undertaking economic evaluations, such as the one presented here.

Attempts to quantify the impact of a CHW intervention, both in terms of costs and outcomes, presents challenges.^45^ Some of these challenges have been addressed, such as including a CHW salary cost in the model. However, other challenges remain, including an estimation of the full cost of epilepsy to the individual and society in rural South Africa. There is a paucity of these data in rural South Africa and, as such, the data used in this study were complemented by expert opinion. As mentioned recently in an article exploring out‐of‐pocket, outpatient costs of epilepsy care in rural South Africa,^46^ further cost‐of‐illness studies are necessary to ascertain the full cost of epilepsy to both patients and society in rural South Africa.

Another challenge is modeling the full benefit of the CHW.^45^ The current study models the health gain anticipated from the introduction of an intervention that aims to improve the epilepsy treatment gap, but fails to take into account individual and social nonhealth benefits. Furthermore, the introduction of CHWs who visit households is likely to have benefits beyond ASM adherence, with a recent South African qualitative study finding that CHWs take on numerous roles^44^ and the potential to positively impact other healthcare needs as well.

The intervention proposed in this analysis aims to reduce the epilepsy treatment gap by improving adherence and does not specifically address the epilepsy diagnostic gap (identifying and diagnosing individuals with epilepsy). Together, the diagnosis and treatment of epilepsy form components of the epilepsy care cascade.^17^ The present analysis builds on earlier work undertaken in rural northeastern South Africa, which found a substantial treatment gap and a much lower diagnostic gap.^17^ As such, the current intervention focuses specifically on improving the treatment gap. That said and, as noted above, all benefits of intervention may not be accounted for in the current model. For example, it is possible that increased community education by CHWs may result in increased community understanding of the role that biomedicine can play in the control of seizures. This, in turn, may reduce stigma and result in more community individuals with epilepsy—previously undiagnosed—being referred for care to local health facilities. Should such a scenario play out, the intervention would likely prove to be even more cost‐effective.

Finally, the health state utility weights used in this study were derived from DWs used in the 2010 GBD study and again in the 2019 study (with the 2010 study presenting weights according to treatment, whereas the 2019 study presented weights according to severity defined by seizure frequency^47^). A utility weight and DW, while similar, are intrinsically different. The utility weight, or QALY, reflects the preference of an individual for health states, whereas the DW reflects the reduction in health due to a disease or condition.^48^ Taking the inverse of the DW can yield an approximate utility value, given that utility values are generally lacking in LMICs.^49^ Both DW and health state utility value calculations now rely on paired comparison questions, which require individuals to rank the health state of two hypothetical individuals with differing health states.^24^ The paucity of contextualized utility values and similar methodologies justify the methodology used in this modeling exercise, although using different values could result in different results.

## 5 CONCLUSION

The introduction of a CHW to monitor ASM adherence in rural South Africa is modeled to be cost‐effective. CHWs are likely to provide a sustainable, local, cost‐effective way of reducing the epilepsy treatment gap in rural South Africa through improved adherence and can, indirectly, benefit other conditions requiring adherence to chronic medication. The use of CHWs to fill a void in the healthcare workforce in sub‐Saharan Africa should be explored as healthcare demands for chronic conditions continue to increase. More generally, the use of economic evaluations to evaluate the cost effectiveness of an intervention or package of interventions, even prior to the implementation of the intervention, can provide decision‐makers and funding bodies with important information to assist with determining their next steps.

## CONFLICT OF INTEREST

The authors declare no competing interests. We confirm that we have read the Journal's position on issues involved in ethical publication and affirm that this report is consistent with those guidelines.

## AUTHOR CONTRIBUTIONS

RGW, FN, LF, CRN, and LL conceptualized and designed the study and analysis. ST and MYB were involved in interpretation of the results. All authors contributed to drafting and editing the manuscript and approved the final version.
